# MiR-3613-5p targets AQP4 to promote the progression of chronic atrophic gastritis to gastric cancer

**DOI:** 10.3389/fphar.2025.1523689

**Published:** 2025-04-04

**Authors:** Jian Bi, Yufen Wang, Yingde Wang

**Affiliations:** ^1^ Department of Gastroenterology, First Affiliated Hospital of Dalian Medical University, Dalian, China; ^2^ Department of Digestive Endoscopy, First Affiliated Hospital of Dalian Medical University, Dalian, China

**Keywords:** chronic atrophic gastritis, miR-3613-5p, gastric cancer, Aquaporin 4, intestinal metaplasia

## Abstract

**Introduction:** Gastric cancer (GC) exhibits high invasiveness, delayed diagnosis, and poor prognosis. Chronic atrophic gastritis (CAG), an initial stage within the Correa cascade, induces gastric mucosal inflammation and atrophy, promoting genetic and epigenetic alterations. MicroRNAs (miRNAs) dysregulation has been implicated in gastric tumorigenesis, yet their specific roles in CAG progression to GC remain unclear.

**Methods:** Using clinical data from the GEO database, we identified miRNAs differentially expressed in gastric mucosa and serum samples from GC patients. Murine CAG models were established through administration of N-methyl-N-nitrosourea (MNU) and high-salt diet (HSD). In vitro functional assays evaluated proliferation and migration after miRNA modulation in gastric cancer cell lines. MiRNA target validation involved luciferase reporter assays.

**Results:** MiR-3613-5p expression was significantly elevated in gastric mucosal and serum samples of GC patients, mucosal tissues of CAG patients, tumor tissues, and human gastric cancer cell lines. Murine models demonstrated increased miR-3613-5p expression in gastric mucosa following MNU and HSD-induced CAG. Functionally, miR-3613-5p overexpression promoted gastric cancer cell proliferation and migration in vitro, whereas silencing miR-3613-5p alleviated pathological gastric mucosal alterations (atrophy, hyperplasia, inflammatory infiltration) in vivo. Mechanistically, miR-3613-5p inhibited Aquaporin 4 (AQP4) expression by directly targeting its 3'UTR.

**Discussion:** Our findings provide the first evidence that miR-3613-5p facilitates CAG progression toward GC via negative regulation of AQP4. These results highlight miR-3613-5p as a promising biomarker and therapeutic target, suggesting antagomiR-3613-5p as a potential novel strategy to prevent gastric carcinogenesis.

## Highlights

• miR-3613-5p is significantly overexpressed in gastric mucosal tissue samples from CAG patients, tumor samples from gastric cancer patients, human gastric cancer cell lines, and the gastric mucosa of CAG mice.
• Overexpression of miR-3613-5p promotes the proliferation and migration of gastric cancer cells, while silencing miR-3613-5p alleviates pathological changes, including atrophy, hyperplasia, and inflammatory accumulation, in the gastric mucosa of CAG mice.
• miR-3613-5p inhibits the expression of the AQP4 gene by binding to its 3′UTR, thereby promoting the progression from CAG to gastric cancer.

## Introduction

Gastric cancer (GC) is a major global health issue, with the highest prevalence observed in East Asia (Sung et al., 2021). It has a high mortality rate due to its aggressive invasiveness, late-stage diagnosis, and poor prognosis (Zheng et al., 2004). Chronic atrophic gastritis (CAG) is a well-established precursor of gastric cancer (Hahn et al., 2024). CAG is characterized by the progressive loss of gastric glandular cells, leading to thinning of the gastric mucosa, with subsequent replacement by fibrous tissue and intestinal metaplasia (Jia et al., 2024). Clinically, CAG often presents with non-specific symptoms, including epigastric discomfort, bloating, and anemia due to impaired absorption of vitamin B12 and iron (Li et al., 2024). The Correa cascade model outlines the sequential progression of gastric carcinogenesis, starting with CAG and advancing through intestinal metaplasia, dysplasia, and ultimately gastric adenocarcinoma (He et al., 2022; Tong et al., 2024). The persistent inflammation and atrophy of the gastric mucosa caused by CAG promote both genetic and epigenetic changes (He et al., 2022). Existing studies identify key signaling pathways, including Wnt/β-catenin, NF-κB, RAS/RAF/MEK/ERK, and PI3K/AKT/mTOR, which promote cell proliferation, migration, and invasion, driving the transition to intestinal metaplasia and dysplasia (Akhavanfar et al., 2023; Guo et al., 2024; Lim et al., 2023). Investigating the factors and specific molecular mechanisms that induce the progression of CAG to gastric cancer is crucial for early diagnosis and intervention.

MicroRNAs (miRNAs) are a class of small, non-coding RNA molecules, typically 18–25 nucleotides in length, that play a critical role in regulating gene expression (Chong, 2024). They primarily function by binding to complementary sequences in the 3′untranslated regions (3′UTR) of target messenger RNAs (mRNAs), leading to mRNA degradation or translational repression (Sun et al., 2024). This post-transcriptional regulation allows miRNAs to fine-tune gene expression in various biological processes, including development, differentiation, apoptosis, and proliferation (Yu et al., 2024). Dysregulation of miRNAs has been implicated in the pathogenesis of numerous diseases, including cancer, cardiovascular diseases, and neurological disorders (Gan et al., 2024; Di Fiore et al., 2024). In gastric cancer (GC), miRNAs play a crucial role in regulating key cancer-related genes and signaling pathways, functioning as oncogenes or tumor suppressors (Alessandrini et al., 2018). For example, miRNA-21 is upregulated in GC and promotes cell proliferation and invasion by targeting PTEN in the PI3K pathway (Motoyama et al., 2010). In contrast, miRNA-375 is downregulated in GC and acts as a protective factor by targeting PDK1, thereby reducing cell viability (Tsukamoto et al., 2010). At least dozens of miRNAs have been shown to play key roles in GC initiation, metastasis, and drug resistance (Christodoulidis et al., 2024). However, the exact role of miRNAs in the transition from chronic atrophic gastritis (CAG) to GC remains unclear. We hypothesize that specific miRNAs play a pivotal role in the progression of CAG to GC by regulating key signaling pathways and gene expression. Identifying these miRNAs could provide novel therapeutic targets for preventing the development and progression of GC.

The ability of miRNAs to simultaneously regulate multiple genes and pathways makes them critical players in disease development and progression. As a result, miRNAs are being explored as potential biomarkers for diagnosis and prognosis, as well as therapeutic targets for novel treatments (Leng et al., 2024; Hu et al., 2024). The main strategies for miRNA-targeted therapy include the use of miRNA mimics and antagomirs. miRNA mimics are synthetic double-stranded RNA molecules designed to restore the function of miRNAs that are downregulated in diseases (Lee et al., 2024). Conversely, antagomirs are single-stranded oligonucleotides that bind to overexpressed miRNAs, preventing them from interacting with their target mRNAs and thereby reducing their pathogenic effects (Rupaimoole and Slack, 2017). miRNA-targeted therapies have shown significant potential in preclinical studies and are being evaluated in clinical trials for diseases such as cancer (Kara et al., 2022). In particular, different types or subtypes of cancer appear to have distinct miRNA expression profiles (Goh et al., 2016). When developing miRNA-targeted therapeutic approaches, it is crucial to identify specific miRNAs associated with tumor subtypes.

Through the analysis of clinical data, we found that miR-3613-5p is significantly upregulated in both the gastric mucosa and serum of gastric cancer patients. The expression of miR-3613-5p is significantly increased in both the gastric mucosa of CAG patients and the tumors of gastric cancer patients. High expression of miR-3613-5p was also observed in human gastric cancer cell lines and the gastric mucosa of CAG mice. Overexpression of miR-3613-5p promotes the proliferation and migration of gastric cancer cells and exacerbates atrophy, hyperplasia, and inflammatory accumulation in the gastric mucosa of CAG mice. We found that miR-3613-5p promotes the progression of CAG to gastric cancer by inhibiting the expression of AQP4. These results identify new potential targets for the early intervention of gastric cancer.

## Materials and methods

### Bioinformatics analysis

We searched the GEO database for non-coding RNA-seq profiles using the keywords “gastric cancer” and “gastritis” from the past 3 years. Raw gene expression profile data and clinical information were available from the GEO database. The following filtering criteria were used: The tissue were obtained from human gastric mucosal and serum, and the number of each group less than 10 were excluded. Finally, non-coding RNA-seq profiles GSE186595 and GSE211692 were obtained. The GSE186595 dataset was used on the GPL20795 platform. The dataset contained 13 gastric mucosa from gastric cancer patients and 20 from healthy controls. The GSE211692 dataset was used on the GPL21263 platform. The dataset contained 1,418 serum microRNA profiles from gastric cancer patients and 5,643 from healthy controls. Furthermore, we also chose GSE130823 dataset to analysis the downregulated genes in gastric cancer carcinogenesis. This dataset used GPL17077 platform, and there are ninety-four biospy samples collected from gastric cancer carcinogenesis and paired controls. DeSeq2 and limma R packages were used to analyzed differentially expressed genes (DEGs) in datasets, and the standard p value <0.05 and logFC >2 was selected as the cut-off standard. For the correlation analysis between AQP4 and miR-3613-5p, we used the GSE224056 dataset from the GEO database, which includes mRNA and miRNA expression profiles from gastric cancer tissues and their corresponding non-tumorous adjacent tissues from five patients. A total of 439 gastric cancer cases with mRNA and miRNA expression profiles were obtained from The Cancer Genome Atlas (TCGA) database (https://www.cancer.gov/ccg/access-data). Pearson correlation analysis between AQP4 and miR-3613-5p was performed on the aforementioned data using R.

### Human tissue specimens

This study included eight gastric mucosal from CAG patients and healthy individuals, as well as six human gastric cancer and adjacent non-tumor tissues. Samples were gathered from the First Affiliated Hospital of Dalian Medical University (Dalian, China) from December 2023 to January 2024. All patients provided informed consent. This study was approved by the First Affiliated Hospital of Dalian Medical University.

### Animals

Mice were bred and maintained at Hubei Academy of Preventive Medicine, Hubei Provincial Center for Disease Control and Prevention. Animal studies were approved by the Laboratory Animal Care and Use Committee of Hubei Provincial Center for Disease Control and Prevention. The experimental procedures followed were in accordance with institutional guidelines. Experiments were performed using 4-week-old wild-type (WT) mice. All experiments on mice were conducted in a C57BL6/N background. Animals were housed under standard conditions and maintained on commercial mouse chow *ad libitum*. The environment was maintained at 22 °C with a 12-h light-12-h dark cycle. N-Nitroso-N-methylurea (MNU, HY-34758, MCE, United States) was added to the drinking water, which the mice were allowed to drink freely, and were gavaged with 1 mL of saturated NaCL solution every 3 days for 12 weeks.

### Cell culture and transfections

The gastric carcinoma cell lines BGC-823, MKN-45, SGC-7901 and the normal gastric mucosal epithelial cell line GES-1 were obtained from the Cell Bank of the Chinese Academy of Sciences (Shanghai, China). These cells were cultured in DMEM (Gibco, United States), supplemented with 10% fetal bovine serum (Gibco, United States) at 37 °C in a humidified incubator with 95% air and 5% CO_2_. Cells were cultured in 6-well plates and transfected when they reached 60%–70% confluence. Agomirs, antagomirs, and their corresponding negative controls (20 nmol) were transfected into the cells using Lipofectamine 3,000 (Invitrogen, United States), following the manufacturer’s instructions. The transfection reagent mixture was incubated with the cells at 37°C for 6 h. After incubation, the medium was replaced with DMEM supplemented with 10% FBS. Cells were collected for subsequent experiments 24 h after transfection. Agomirs and antagomirs and their negative controls were obtained from GenePharma (Suzhou, China). The AgomiR-3613-5p sequence was 5′- UGU​UGU​ACU​UUU​UUU​UUU​GUU​C-3′. The AgomiR-NC sequence was 5′- AUC​UAU​ACU​UUG​UUU​UUC​UUC​U-3′. The AntagomiR-3613-5p sequence was 5′- GAA​CAA​UUU​UUU​UAC​ACA​A-3′. The Antagomir-NC sequence was 5′- CUU​AGU​UUU​UAU​CAU​UCA​A -3′.

### Histology

Tissues were harvested from euthanized animals and immediately fixed in 4% paraformaldehyde fixative for 24 h at room temperature. After fixation, the samples were dehydrated through a graded ethanol series (70%, 80%, 95%, and 100%) and embedded in paraffin. Sections of 4 μm thickness were cut using a microtome and mounted on glass slides. To perform Hematoxylin and Eosin (H&E) staining, slides were deparaffinized with xylene and rehydrated through a descending ethanol series. The tissue sections were then stained with hematoxylin for 5 min, rinsed, and differentiated in 1% hydrochloric acid alcohol. Eosin staining was performed for 2 min, followed by dehydration, clearing, and mounting. Slides were observed under microscope (*Nikon*, Japan) for morphological analysis.

### ELISA

The blood samples were left at room temperature for 2 h, and then centrifuged at 1,000°g at 4°C for 20 min. The supernatant was collected, and the levels of IL-6 (Mouse IL-6 ELISA Kit, 98027ES96, Yeasen Biotechnology, China), TNF-α (Mouse TNF-α ELISA Kit, 98029ES96, Yeasen Biotechnology, China) in the serum of mice were measured by ELISA using the corresponding commercial kits. The operation was conducted strictly according to the manufacturer’s instructions. The standards were diluted according to the multiplicative dilution method, and a standard curve was prepared for each assay. Three replicate wells were set up for each sample, and each experiment was repeated three times.

### Cell proliferation assay

Cells were cultured on 12-well slides for 24 h, fixed with 4% paraformaldehyde, permeabilized with 0.5% Triton X-100, and incubated with Ki-67 primary antibody (1:500, 27309-1-AP, Proteintech, China) at 4 °C overnight. After three washes with Tris Buffered Saline-Tween20, the sections were incubated with fluorescent secondary antibody (A23420, Abbkine, United States) for 1 h. Nuclei were stained with DAPI (P0131, Beyotime, China). Ki-67-positive cells and total cell counts were determined in 12 fields of view (×20 objective) from three replicate wells using the ImageJ analysis software. The Ki-67 index was calculated as the ratio of Ki-67-positive cells to the total number of cells.

### Wound healing assay

At ∼90% confluence, a scratch was made in the center of each well using the tip of a 200 μL pipette. Subsequently, the cells were cultured in serum-free medium. Images of the wounds were captured at 0 and 24 h by an Olympus CKX53 inverted microscope at ×4 magnification (Olympus Corporation) to record the scratch width marking the scratch location. The migration index was calculated to evaluate the cell migration capacity. Migration index = (A0h - A24h)/A0h. Scratch area (Area, A) was measured by ImageJ software (version 1.52a; National Institutes of Health). Every experiment was repeated 3-times.

### Luciferase constructs and transfection

The 3′-UTR of AQP4 was amplified by PCR and subcloned into the dual-luciferase reporter vector pGL3 (Promega, United States) at Mlul and Xhol sites, termed Luc-AQP4-WT. The forward primer was 5′-CCG​AGC​TCT​TAC​GCG​CTA​GTT​GAG​TCC​TGG​CTT​T-3′. The reverse primer was 5′-GAT​CGC​AGA​TCT​CGA​GAC​AAG​TAA​GTG​AGT​CAG​TAA​C-3′. The mutant vectors with point mutations in miR-3613-5p binding sites were synthesized using the QuikChange Site-Directed Mutagenesis Kit (Stratagene, United States), termed Luc-AQP4-MUT. HEK293T cells transfected with AgomiR-3613-5p and AntagomiR-3613-5p and their negative controls, with pGL3, Luc-AQP4-WT or Luc-AQP4-MUT plasmid. Renilla luciferase was co-transfected for the purposes of normalization. Cells were harvested 24 h after transfection and assayed for firefly luciferase and Renilla luciferase activities by Dual Luciferase Reporter Gene Assay Kit (KTA8010, Abbkine, United States).

### RT-PCR

Total RNA was extracted using the RNAiso Plus (9,109, Takara, Japan) method according to the manufacturer’s protocol and reverse transcribed into cDNA using a cDNA synthesis kit (AT311, TransGen Biotech, China). For mRNA detection, gene expression was analyzed by the Tip Green qPCR SuperMix kit (AQ142, TransGen Biotech, China), and the results were normalized to GAPDH expression. For miRNA detection, cDNA was synthesized with the Mir-X™ miRNA First Strand Synthesis Kit (Takara), and subsequent qPCR analysis was performed using the Tip Green qPCR SuperMix kit (AQ142, TransGen Biotech, China). U6 snRNA was used as the endogenous control to normalize miRNA expression. Primers (synthesized by Sangon Biotech, China) for miR-3613-5p, PTGER3, HSPB7, AQP4, ERBB4, PAQR5, U6 snRNA and Gapdh were as follows.

**Table:** 

miR-3613-5p	Forward primer	5′-UGU​UGU​ACU​UUU​UUU​UUU​GUU​C-3′
	reverse primer	5′-GTGCAGGGTCCGAGGT -3’
PTGER3	forward primer	5′-CGC​CTC​AAC​CAC​TCC​TAC​ACA-3′
	reverse primer	5′-ATC​CGC​AAT​CCT​CGC​CAG​AC-3’
HSPB7	forward primer	5′-CAC​CAC​CTC​CAA​CAA​CCA​CAT​C-3′
	reverse primer	5′-TGG​CAC​TTG​TGA​GCG​AAG​GT-3’
AQP4	forward primer	5′-AGC​ATC​GCC​AAG​TCT​GTC​TTC​T-3′
	reverse primer	5′-GAG​ACC​ATG​ACC​AGC​GGT​AAG​A-3’
ERBB4	forward primer	5′-GAA​CAG​CAG​TAC​CGA​GCC​TTG-3′
	reverse primer	5′-CGA​ACA​GAC​CGC​AGG​AAG​GA-3’
PAQR5	forward primer	5′-CCT​GGA​CTA​TGG​TGC​CGT​CAA-3′
	reverse primer	5′-GCC​TGT​GCT​GAG​GAT​GGT​GTT-3’
U6 snRNA	forward primer	5′-CGC​TTC​GGC​AGC​ACA​TAT​AC-3′
	reverse primer	5′-TTC​ACG​AAT​TTG​CGT​GTC​AT-3’

### Protein extraction and Western blot

Tissues and cells were lysed in RIPA Lysis buffer (Bioss, China) supplemented with cOmplete Protease Inhibitor EASYpacks (Roche, Switzerland) on ice for 30 min. Protein fractions were collected by centrifugation at 13,000 g at 4°C for 15 min. Protein samples were separated by 10% SDS–PAGE and transferred to nitrocellulose membranes. The membranes were blocked with 5% skimmed milk and incubated with the primary antibodies overnight. Antibodies used were: Tubulin (1:5,000, ABL1030, Abbkine, China), AQP4 (1:1,000, 16473-1-AP, Proteintech, China).

### Statistical analysis

For statistical analysis, all quantitative data are presented as the mean ± SEM. Statistical analysis for comparison of two groups was performed using two-tailed unpaired Student’s t-test. Statistical differences among groups were analyzed by 1-way analysis of variance (ANOVA) or 2-way ANOVA (if there were two factor levels), followed by Bonferroni’s *post hoc* test to determine group differences in the study parameters. Before ANOVA, we firstly test the homogeneity among variances. If it shows the variances are unequal, we then use Welch’s ANOVA for 1-way analysis and the ordinary 2-way ANOVA for 2-way analysis after log-transformation. Pearson correlation coefficients was performed to assess the correlation between two variables. All statistical analyses were performed with GraphPad PrismV7 (GraphPad Prism, United States) and SPSS 23.0 software (SPSS, United States). Differences were considered significant at *P < 0.05, **P < 0.01, ***P < 0.001.

## Results

### Result 1: miR3613-5p is significantly overexpressed in human CAG and gastric cancer tissues

**FIGURE 1:**
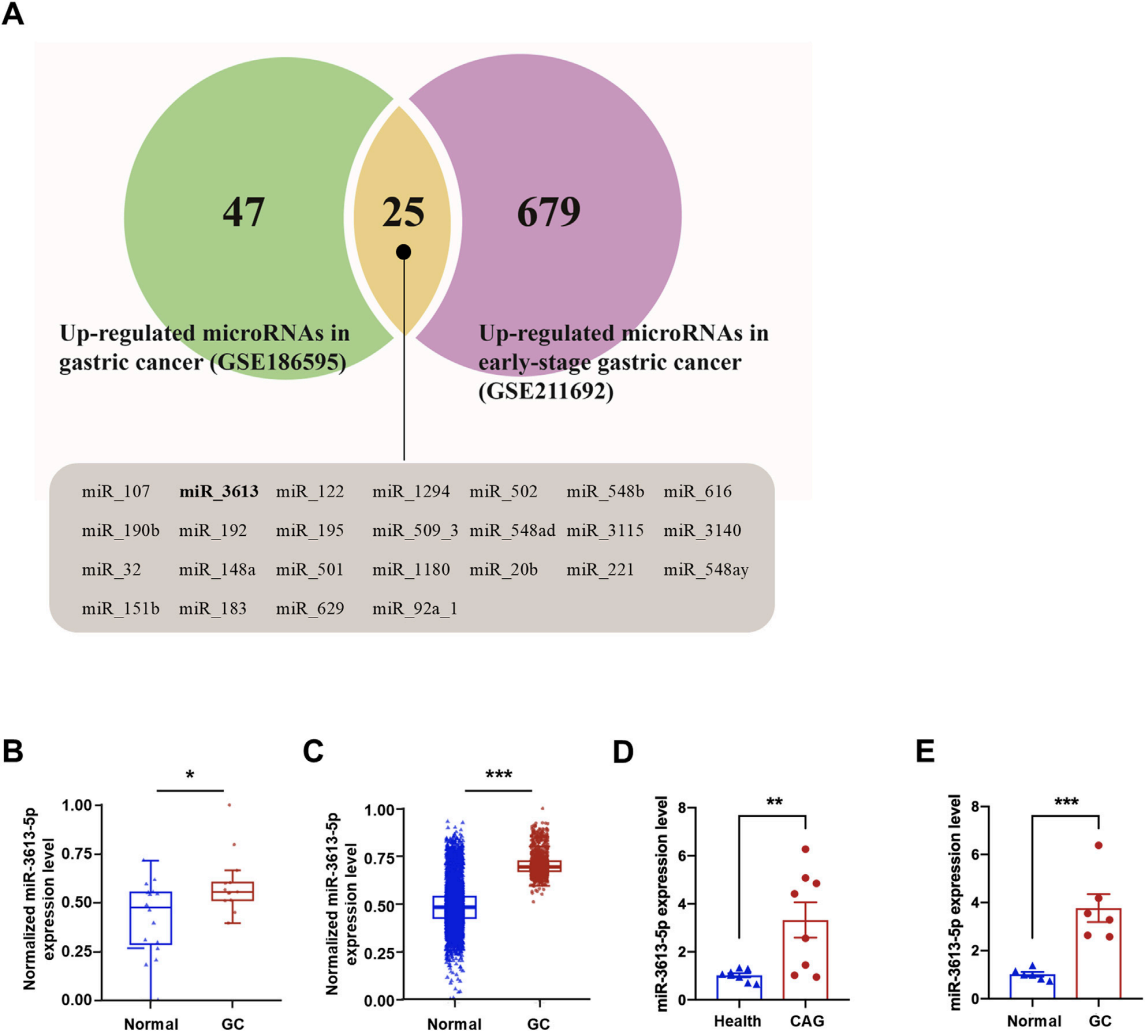
miR3613-5p is significantly overexpressed in human CAG and gastric cancer tissues. **(A)** Venn diagram of significantly upregulated miRNAs in the GSE186595 and GSE211692 datasets. A total of 72 differentially expressed genes (DEGs) for microRNAs were significantly upregulated in the gastric mucosal of gastric cancer patients compared to healthy controls in the GSE186595 dataset (green), while 704 DEGs were significantly upregulated in the serum of early gastric cancer patients compared to healthy controls in the GSE211692 dataset (purple). There are 25 microRNAs that are differentially expressed in both datasets (yellow), all of which are displayed below, including miR-3613. **(B, C)** Box plot of miR-3613-5p expression level in GSE186595 **(B)** and GSE211692 **(C)** dataset. **(D)** Relative expression levels of miR-3613-5p in gastric mucosal from CAG patients and healthy individuals (n = 8). **(E)** Relative expression levels of miR-3613-5p in gastric cancer and adjacent non-tumor tissues (n = 6). Data are presented as mean ± SEM. Statistical analysis is performed using a Student’s t-test and statistical significance is shown as *p < 0.05, **p < 0.01, and ***p < 0.001.

We searched the GEO database for non-coding RNA-seq profiles from the past 3 years and obtained the GSE186595 and GSE211692 datasets. The GSE186595 dataset includes microRNA profiles from gastric mucosa tissues of 13 gastric cancer patients and 20 healthy controls. The GSE211692 dataset contains serum microRNA profiles from 1,418 early gastric cancer patients and 5,643 healthy controls. A total of 72 and 704 differentially expressed genes (DEGs) were identified in the GSE186595 (green) and GSE211692 (purple) datasets, respectively (Figure 1A). Among the two datasets, 25 miRNAs are commonly differentially expressed, including miR-3613. In both the GSE186595 and GSE211692 datasets, the expression of miR-3613-5p was significantly upregulated (Figures 1B,C). In recent years, miR-3613-5p has emerged as an important target in cancer research and has been shown to play a regulatory role in various tumor types (Luo et al., 2022; Zhan et al., 2021; Qin et al., 2019). Therefore, we aim to understand the role of miR-3613-5p in the progression of CAG to gastric cancer. We clinically collected gastric mucosa samples from eight patients with chronic atrophic gastritis (CAG) and healthy individuals to assess the expression of miR-3613-5p (ethics approval No. PJ-KS-KY-2023–528). Our findings revealed a significant upregulation of miR-3613-5p in the gastric mucosa of CAG patients (Figure 1D). Additionally, we examined miR-3613-5p expression in gastric cancer and adjacent non-tumor tissues. In 6 cases of gastric cancer, the expression level of miR-3613-5p was significantly higher than in the corresponding adjacent non-tumor tissues (Figure 1E). These results suggest that miR-3613-5p expression is increased in both CAG and gastric cancer tissues.

### Result 2: miR3613-5p is significantly overexpressed in gastric cancer cell lines and the gastric mucosa of CAG mice

**FIGURE 2:**
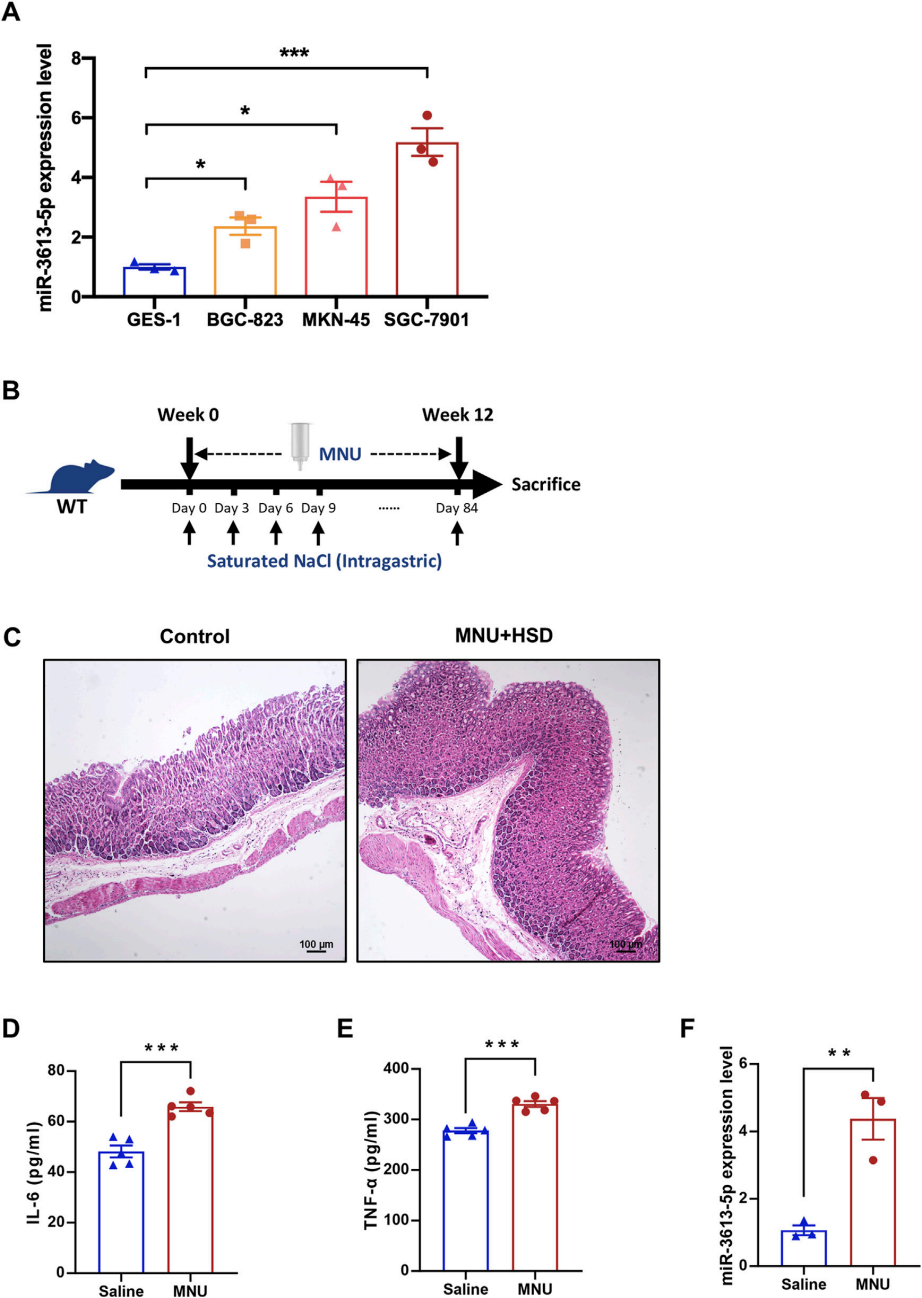
miR3613-5p is significantly overexpressed in gastric cancer cell lines and the gastric mucosa of CAG mice. **(A)** Relative expression levels of miR-3613-5p in human gastric cancer cell lines (BGC-823, MKN-45, SGC-7901) and the normal gastric mucosal epithelial cell line GES-1. **(B)** Schematic diagram of the modeling method for CAG mice. MNU was added to the drinking water for the mice to drink freely, and a 1mL saturated NaCl solution was administered by gavage every 3 days, continuing for 12 weeks. **(C)** Representative H&E staining images of gastric mucosal slices from control and MNU + HSD mice after 12 weeks of induction. **(D-E)** Levels of IL-6 and TNF-α in the serum of control and MNU + HSD mice detected by ELISA (n = 5). **(F)** Relative expression levels of miR-3613-5p in the gastric mucosa of control and MNU + HSD mice (n = 5). Data are presented as mean ± SEM. Statistical analysis is performed using a Student’s t-test and statistical significance is shown as *p < 0.05, **p < 0.01, and ***p < 0.001.

We examined the expression of miR-3613-5p in three human gastric cancer cell lines with varying degrees of differentiation, using the normal gastric mucosal epithelial cell line GES-1 as a control. RT-PCR results revealed a significant increase in miR-3613-5p expression across all three gastric cancer cell lines, with the highest expression observed in the moderately differentiated SGC-7901 cell line (Figure 2A). Subsequently, we induced a mouse model of CAG using N-Methyl-N-nitrosourea (MNU) combined with a high-salt diet (HSD) (Figure 2B). MNU is a potent chemical carcinogen that induces apoptosis or necrosis of gastric mucosal epithelial cells, triggering abnormal proliferation and atypical hyperplasia of gastric epithelial cells. A high-salt diet is considered one of the key risk factors for CAG and gastric cancer, as it increases the osmotic pressure of gastric contents, directly damaging gastric mucosal cells, leading to shedding, necrosis, and inflammatory responses in the mucosal epithelial cells. After 12 weeks of induction, the gastric mucosal morphology of the control mice remained normal, with gastric epithelial cells showing typical glandular structures, and the gastric pits comprising approximately one-third of the gland thickness. In contrast, the gastric mucosa of the induced mice exhibited severe atrophy, loss of intrinsic glands, hyperplasia-like changes, and the transformation of chief cells into mucous cells (Figure 2C). Significant infiltration of inflammatory cells was observed in the mucosal base and lamina propria. Consistently, the serum levels of inflammatory cytokines interleukin-6 (IL-6) and tumor necrosis factor-α (TNF-α) were significantly increased (Figures 2D,E). Pathological and serological test results confirmed that the mice induced with MNU and a high-salt diet for 12 weeks developed the pathophysiological characteristics of CAG. Notably, the expression of miR-3613-5p was significantly increased in the gastric mucosa of the induced mice (Figure 2F).

### Result 3: miR-3613-5p promotes the proliferation and migration of gastric cancer cells

**FIGURE 3:**
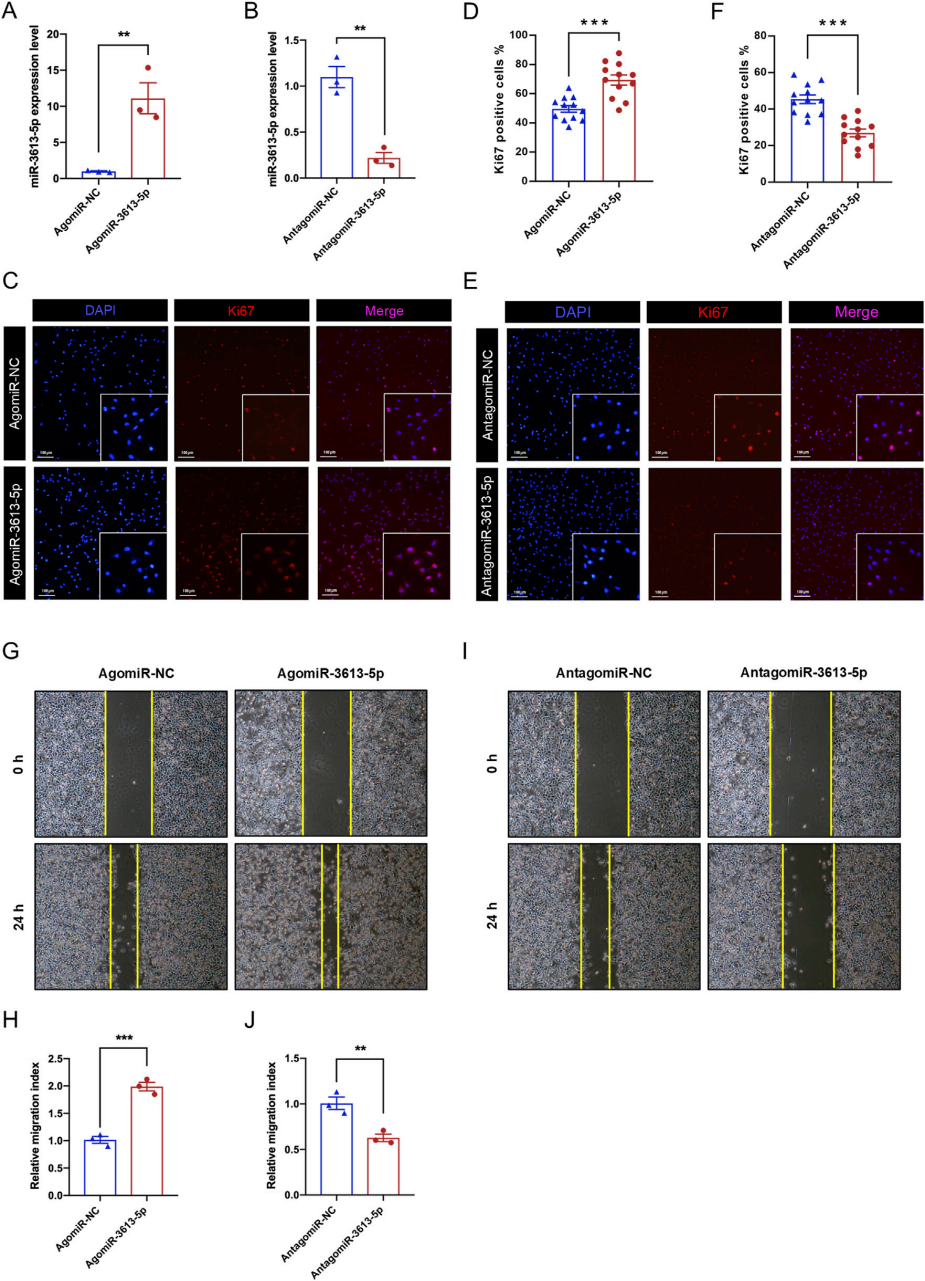
miR-3613-5p promotes the proliferation and migration of gastric cancer cells. **(A–B)** Relative expression levels of miR-3613-5p after transfection of AgomiR-3613-5p and AntagomiR-3613-5p in SGC-7901 cell line. **(C, E)** Representative images of Ki-67 immunofluorescence staining after transfection of AgomiR-3613-5p and AntagomiR-3613-5p in SGC-7901 cell line. **(D, F)** Statistical analysis of the percentage of Ki-67 positive cells after transfection of AgomiR-3613-5p and AntagomiR-3613-5p. **(G, I)**. Representative images from the cell wound healing assay after transfection of AgomiR-3613-5p and AntagomiR-3613-5p. **(H, J)** Statistical analysis of the wound healing assay. The migration index was calculated to evaluate the cell migration capacity. Migration index = (A0h - A24h)/A0h. Scratch area (Area, A) was measured by ImageJ software (version 1.52a; National Institutes of Health). Every experiment was repeated 3 times. Data are presented as mean ± SEM. Statistical analysis is performed using a Student’s t-test and statistical significance is shown as *p < 0.05, **p < 0.01, and ***p < 0.001.

Since the SGC-7901 cell line exhibited the highest expression of miR-3613-5p among the three gastric cancer cell lines tested, we selected it for small molecule delivery and functional assays. We used a synthetic small RNA fragment, agomiR-3613-5p, to mimic the function of endogenous miR-3613-5p, and antagomiR-3613-5p to specifically inhibit its function. RT-PCR results showed that agomiR-3613-5p could increase miR-3613-5p expression by more than tenfold in gastric cancer cells, while antagomiR-3613-5p significantly reduced its expression (Figures 3A,B). During the progression from CAG to gastric cancer, chronic inflammation progressively accumulates (Mustapha et al., 2014). The levels of cytokines, such as IL-6 and TNF-α, are elevated, stimulating cell cycle progression and preventing apoptosis, thereby allowing cancer cells to proliferate uncontrollably (Browning et al., 2018; Ji et al., 2014). Ki-67 is a cell proliferation marker that is specifically expressed during the cell cycle. Immunofluorescence staining revealed an increase in the number of Ki-67-positive nuclei after agomiR-3613-5p delivery, with statistical analysis showing a significantly higher percentage of Ki-67-positive cells compared to the negative control (NC) (Figures 3C,D). Conversely, the antagomiR-3613-5p inhibitor significantly reduced the percentage of Ki-67-positive cells (Figures 3E,F). These results suggest that miR-3613-5p promotes the proliferation of gastric cancer cells. The scratch assay results showed that overexpression of miR-3613-5p significantly accelerated the wound healing rate of gastric cancer cells (Figures 3G,H), while inhibition of miR-3613-5p expression markedly slowed the healing process (Figures 3I,J). These findings indicate that miR-3613-5p promotes the migration of gastric cancer cells.

### Result 4: Overexpression of miR-3613-5p promotes the progression from CAG to gastric cancer in mice

**FIGURE 4:**
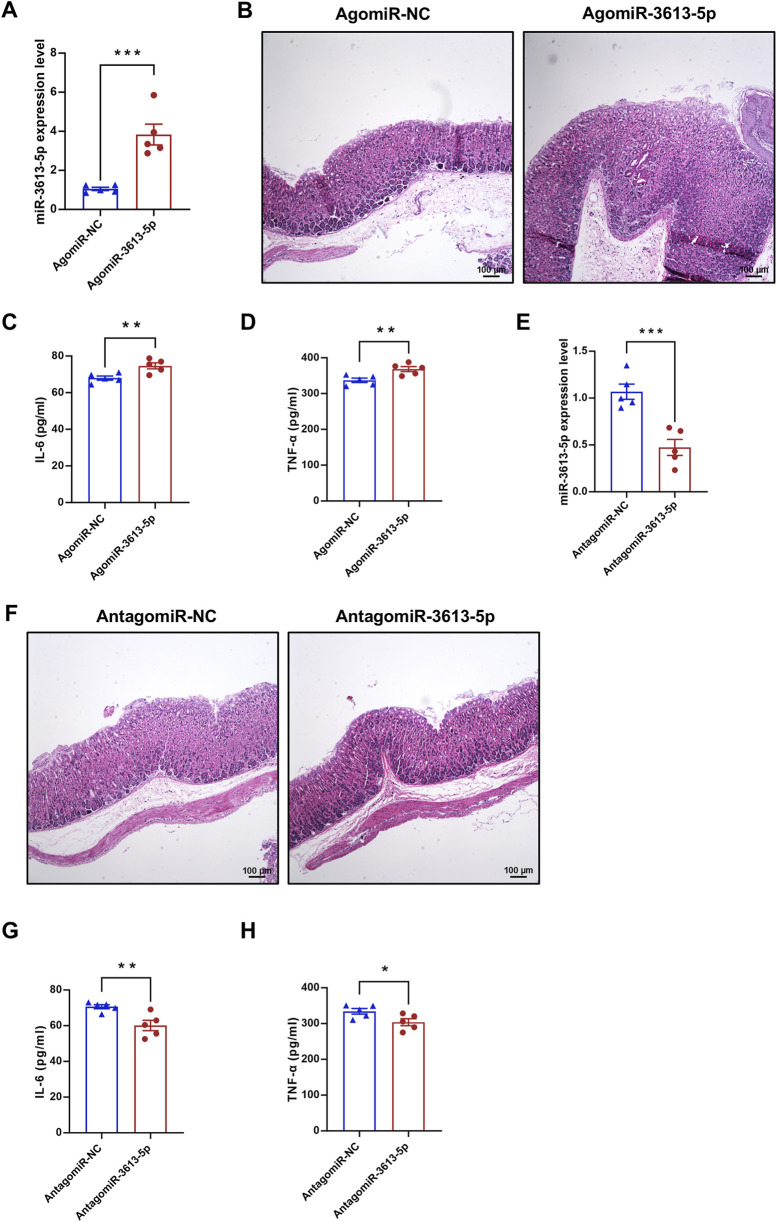
Overexpression of miR-3613-5p promotes the progression from CAG to gastric cancer in mice. **(A, E)** Relative expression levels of miR-3613-5p in gastric mucosa of CAG mice after tail vein injection of AgomiR-3613-5p or AntagomiR-3613-5p. **(B, F)** Representative images of H&E staining of mouse gastric mucosal slices. **(C, G)** Levels of IL-6 in mouse serum detected by ELISA (n = 5). **(D, H)** Levels of TNF-α in mouse serum detected by ELISA (n = 5). Data are presented as mean ± SEM. Statistical analysis is performed using a Student’s t-test and statistical significance is shown as *p < 0.05, **p < 0.01, and ***p < 0.001.

During the final month of CAG model induction in mice, AgomiR-3613-5p was administered *via* tail vein injection to overexpress miR-3613-5p *in vivo* (Figure 4A). Pathological analysis of the gastric mucosa revealed that, compared to AgomiR-NC, AgomiR-3613-5p exacerbated atrophy and hyperplasia of the gastric mucosal intrinsic glands, with intestinal metaplasia and the presence of goblet cells (Figure 4B). Consistently, serum levels of IL-6 and TNF-α significantly increased with miR-3613-5p overexpression, indicating that miR-3613-5p promotes the progression from CAG to gastric cancer in mice (Figures 4C,D). Subsequently, we suppressed miR-3613-5p expression in mice using AntagomiR-3613-5p (Figure 4E) and observed a significant reduction in glandular atrophy, with partial recovery in gland morphology and decreased hyperplasia (Figure 4F). Treatment with AntagomiR-3613-5p also significantly decreased serum levels of IL-6 and TNF-α (Figures 4G,H). This suggests that inhibiting miR-3613-5p expression can alleviate mucosal atrophy, hyperplasia, and inflammatory accumulation in CAG in mice.

### Result 5: miR-3613-5p inhibits the expression of the AQP4 gene by binding to its 3′UTR

**FIGURE 5:**
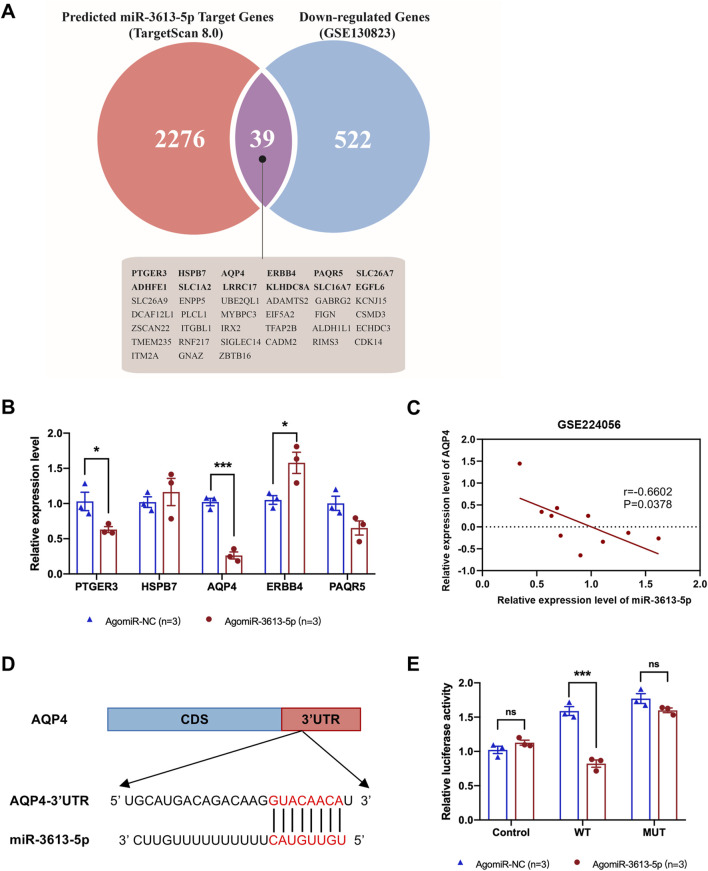
miR-3613-5p inhibits the expression of the AQP4 gene by binding to its 3′UTR. **(A)** The Venn diagram illustrates the overlap between the target genes predicted by TargetScan 8.0 (red) and the downregulated genes in the gastric mucosa of gastric cancer patients from the GSE130823 dataset (blue). The 39 presumed target genes in the intersection are listed below, including AQP4. **(B)** Relative mRNA expression levels of PTGER3, HSPB7, AQP4, ERBB4, and PAQR5 in SGC-7901 cell line after transfection with AgomiR-NC and AgomiR-3613-5p. **(C)** Pearson correlation coefficient between AQP4 and miR-3613-5p. Data from GSE224056. **(D)** Predicted binding sites of miR-3613-5p in the 3′ UTR of the AQP4 gene according to TargetScan 8.0. **(E)** Relative luciferase activity after co-transfection of SGC-7901 cells with pGL3, pGL3-AQP4-WT, pGL3-AQP4-MUT, as well as AgomiR-NC and AgomiR-3613-5p. Data are presented as mean ± SEM. Statistical analysis is performed using a Student’s t-test and statistical significance is shown as *p < 0.05, **p < 0.01, and ***p < 0.001.

The GSE130823 dataset includes gene expression data from 94 gastric cancer tumor tissues and paired control biopsy samples. From this dataset, we identified genes significantly downregulated in gastric cancer and intersected them with miR-3613-5p predicted target genes from TargetScan 8.0, resulting in 39 potential target genes (Figure 5A). Based on differential expression significance, we measured the expression of the top five genes by RT-PCR in the SGC-7901 cell line and found that AQP4 mRNA was the most significantly downregulated (Figure 5B). For external validation, we used the GSE224056 dataset, which includes whole transcriptome sequencing data from gastric cancer tissues and their corresponding adjacent non-tumorous tissues from five patients. By integrating gene expression data with microRNA profiles, we found a negative correlation between miR-3613-5p and AQP4 expression (Figure 5C). A similar trend in AQP4 expression was observed in the Cancer Genome Atlas (TCGA) database (Supplementary Figure S1). We identified the predicted binding sites of miR-3613-5p in the 3′ UTR of the AQP4 gene and introduced mutations at these sites (Figure 5D). We verified the effect of miR-3613-5p on AQP4 gene expression using dual-luciferase reporter assays. Overexpression of miR-3613-5p significantly inhibited expression of the wild-type (WT) AQP4 gene, but had no effect on the AQP4 gene with mutated binding sites (MUT) (Figure 5E). This suggests that miR-3613-5p inhibits AQP4 gene expression by binding to its 3′ UTR.

### Result 6: miR-3613-5p promotes the progression from CAG to gastric cancer by inhibiting the expression of AQP4

**FIGURE 6:**
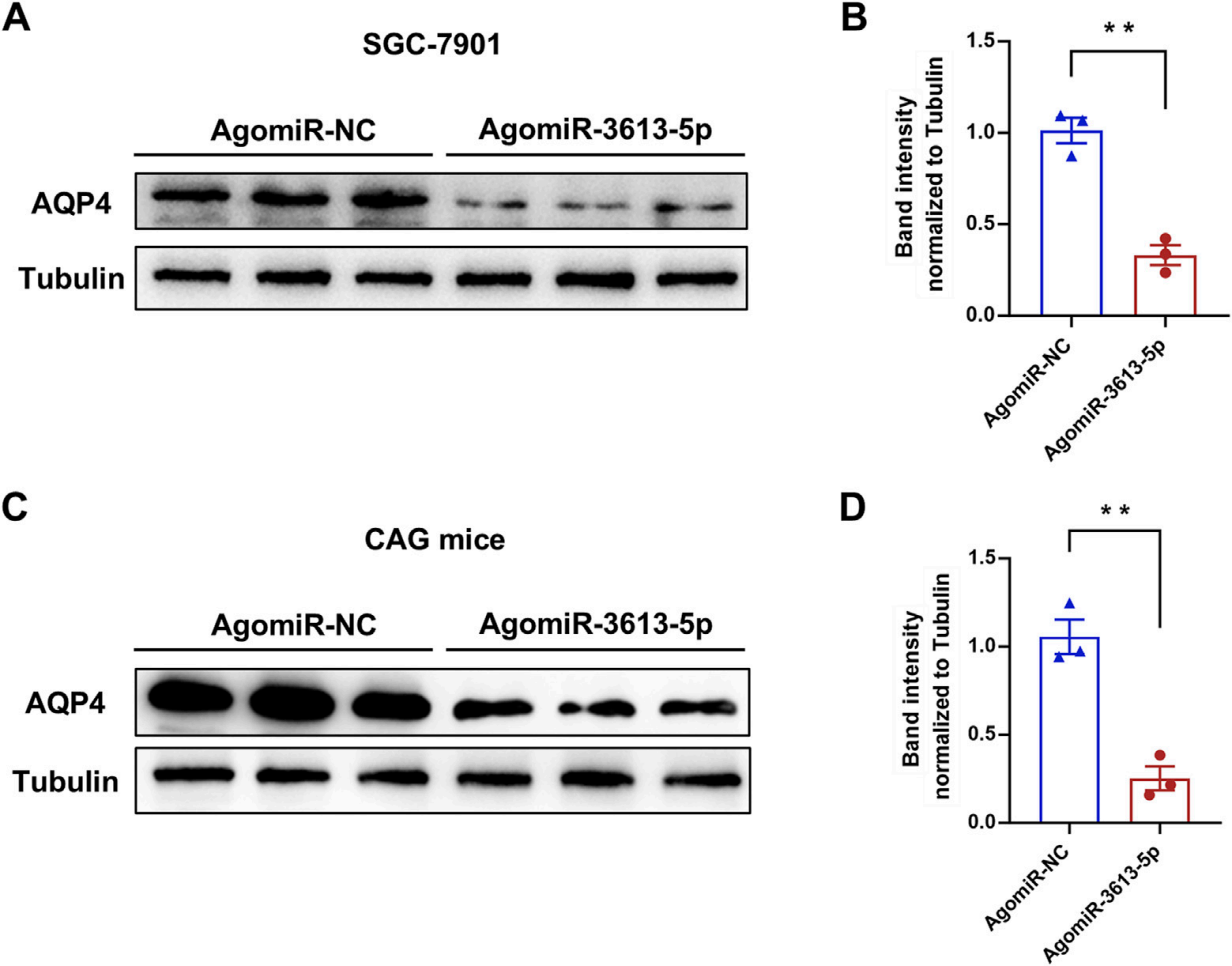
miR-3613-5p promotes the progression from CAG to gastric cancer by inhibiting the expression of AQP4. **(A)** Protein levels of AQP4 after transfection of AgomiR-NC or AgomiR-3613-5p in SGC-7901 cells. **(B)** Quantification of protein expression in **(A)**. **(C)** Protein levels of AQP4 in the gastric mucosa 1 month after tail vein injection of AgomiR-NC or AgomiR-3613-5p in MNU + HSD mice. **(D)** Quantification of protein expression in **(C)** Data are presented as mean ± SEM. Statistical analysis is performed using a Student’s t-test and statistical significance is shown as *p < 0.05, **p < 0.01, and ***p < 0.001.

Aquaporins (AQPs) are integral membrane proteins that regulate the transport of water and small molecules across cell membranes. Thirteen types of AQPs (AQP0–AQP12) have been identified in humans, playing a crucial role in maintaining water homeostasis across various tissues, including the gastrointestinal tract (Nagaraju et al., 2016). In gastric cells, AQPs are involved in regulating gastric acid secretion and maintaining epithelial integrity. Among these, AQP4 is highly expressed in the chief and parietal cells of the gastric mucosa (Misaka et al., 1996). Parietal cells, which secrete HCl, rely on AQP4 to facilitate water entry into the glands, aiding in the dilution and secretion of hydrochloric acid. Dysregulation of AQP4 expression may lead to altered acid secretion and gastric mucosal damage, potentially contributing to ulcer formation (Demitrack et al., 2012). After overexpressing miR-3613-5p in the SGC-7901 cell line, we observed a significant decrease in AQP4 protein levels (Figures 6A,B). Similarly, in CAG mice, overexpression of miR-3613-5p led to a significant decrease in AQP4 protein levels as well (Figures 6C,D). These results confirm that AQP4 is a direct target gene of miR-3613-5p. AQP4 is responsible for water transport in gastric mucosal cells (Misaka et al., 1996). By inhibiting AQP4 expression, miR-3613-5p promotes the progression from CAG to gastric cancer.

## Discussion

In recent years, miR-3613-5p has garnered attention as a potential regulatory factor in tumor biology, with studies suggesting its involvement in the development of various cancers. For example, Qin et al. identified miR-3613-5p as a negative prognostic indicator for hepatocellular carcinoma through a comprehensive analysis of miRNA expression profiles from The Cancer Genome Atlas (TCGA), and KEGG enrichment analysis indicated that it may contribute to tumorigenesis by regulating several signaling pathways, including PI3K/AKT and MAPK(28). Xu et al. found that miR-3613-5p is highly expressed in HCC and can regulate the expression of the tumor marker KMO (Xu et al., 2023). Mohsen et al. reported high expression of miR-3613-5p in renal clear cell carcinoma (RCC) tissues through bioinformatics analysis, where it was associated with clinical parameters such as pathological staging and histological grading (Ahmadi et al., 2023). In a clinical model of RCC constructed by Zhang et al., miR-3613-5p emerged as a prognostic indicator (Zhan et al., 2021). Luo et al. discovered that miR-3613-5p is upregulated in extracellular vesicles of breast cancer and regulates progression by targeting the tumor suppressor gene PTEN, enhancing resistance to doxorubicin (Luo et al., 2022). Fehmida et al. reported significantly elevated miR-3613-5p expression in tumor tissues of seven early-stage and 26 late-stage gastric cancer patients from a Saudi population (Bibi et al., 2016). However, the role of miR-3613-5p in gastric cancer has not been fully explored. Despite this, studies across various cancers confirm that miR-3613-5p is involved in regulating key processes such as cell proliferation, apoptosis, and migration, suggesting its potential role in the occurrence and progression of gastric cancer. Our research demonstrates that miR-3613-5p is significantly upregulated in gastric cancer tissue samples, and its overexpression promotes the proliferation and migration of gastric cancer cells.

Aquaporins (AQPs) are a family of small integral membrane proteins that facilitate the transport of water and small molecules across biological membranes. They play essential roles in maintaining water balance across various tissues, including the kidneys, brain, lungs, and gastrointestinal tract (Nagaraju et al., 2016). AQPs are also emerging as potential diagnostic and therapeutic targets for gastrointestinal cancers, influencing tumorigenesis by regulating cancer cell proliferation, migration, and angiogenesis (Xia et al., 2017). Among the AQPs, AQP4 is primarily expressed in the membranes of chief and parietal cells in the gastric mucosa and is believed to contribute to gastric acid secretion. Parietal cells secrete HCl, and the transport of water is crucial for this process. AQP4 facilitates water entry into the gastric glands, essential for diluting and secreting hydrochloric acid. Dysregulation of AQP4 in the stomach may be linked to abnormal gastric acid secretion and the formation of ulcers (Misaka et al., 1996). In gastric cancer, AQP4 expression is typically decreased compared to normal gastric mucosa (Shen et al., 2010). This downregulation correlates with enhanced tumor invasiveness, poor prognosis, and increased cancer cell migration. Recent studies suggest that AQP4 downregulation may facilitate gastric cancer progression by promoting an inflammatory microenvironment conducive to tumor growth. For example, LINC00629 upregulates AQP4 expression by binding to miR-196b-5p, suppressing gastric cancer cell proliferation and migration (Li et al., 2020). Our findings further suggest that miR-3613-5p negatively regulates AQP4, exacerbating gastric mucosal inflammation and intestinal metaplasia, thus accelerating gastric cancer cell proliferation and migration. This implies that AQP4 may serve a protective role in gastric cancer development, highlighting its potential as a therapeutic target.

The gastric mucosa is regularly exposed to various inflammatory stimuli, such as *Helicobacter pylori* infection, oxidative stress, and gastric acid imbalance, all of which can lead to chronic inflammation (Yang et al., 2021; Butcher et al., 2017). AQP4 is involved in water transport and cellular homeostasis, contributing to the maintenance of mucosal integrity under normal conditions (Misaka et al., 1996). However, when AQP4 expression is downregulated, the gastric epithelium becomes more vulnerable to inflammatory damage, triggering cytokine signaling, particularly the activation of IL-6 and TNF-α. IL-6, a pro-inflammatory cytokine, plays a crucial role in tumorigenesis by promoting cell survival, proliferation, and immune evasion. Elevated levels of IL-6 are commonly observed in gastric cancer tissues and correlate with poor prognosis (Sánchez-Zauco et al., 2017). Our results show that overexpression of miR-3613-5p significantly increases IL-6 levels in the serum of CAG mice. The loss of AQP4 may amplify local IL-6 concentrations in CAG by promoting the activation of inflammatory cells, such as macrophages and neutrophils. IL-6 activates the JAK/STAT signaling pathway, which enhances cancer cell proliferation and resistance to apoptosis (Wu et al., 2025; Zhao et al., 2016). In gastric cancer, TNF-α contributes to angiogenesis, immune suppression, and cancer cell migration (Ji et al., 2014). In our study, serum levels of TNF-α were significantly elevated in mice with overexpression of miR-3613-5p. The downregulation of AQP4 may increase TNF-α expression in the gastric mucosa by activating the NF-κB pathway, further exacerbating inflammation and promoting cancer cell proliferation and migration (Kwon et al., 2012).

Chronic atrophic gastritis (CAG) is a precancerous lesion and is considered a critical step in the Correa cascade model of gastric carcinogenesis. The progression from CAG to gastric cancer is influenced by multiple factors, including persistent inflammation, *H. pylori* infection, as well as genetic and epigenetic alterations (He et al., 2022). Intestinal metaplasia, the next stage in the Correa cascade, involves the replacement of gastric epithelial cells with intestinal-type cells. While this is considered an adaptive response to chronic inflammation, it significantly increases the risk of cancer. The underlying molecular mechanisms of this transformation involve key signaling pathways, including Wnt/β-catenin, EGFR, and Notch (Sugano et al., 2023). High-grade dysplasia is regarded as a precursor to invasive gastric cancer. At this stage, a considerable accumulation of genetic mutations and epigenetic changes occurs, driving the tissue toward malignant transformation. In our study, we found that overexpression of miR-3613-5p triggers immune responses by releasing pro-inflammatory cytokines such as IL-1β and TNF-α. Additionally, it regulates abnormal signaling pathways in gastric epithelial cells, leading to glandular atrophy and intestinal metaplasia, which in turn promote the proliferation and migration of gastric cancer cells. MiR-21 and miR-155 have been widely applied as biomarkers for early gastric cancer detection, and miR-3613-5p may also emerge as a promising new diagnostic target for assessing gastric cancer risk (Farasati Far et al., 2023).

While our study offers valuable insights into the role of miR-3613-5p in the progression from CAG to gastric cancer, there are several limitations to consider. First, the use of mouse models, though informative, may not fully capture the complexity of human gastric cancer. Additionally, the lack of long-term clinical validation means that the therapeutic potential of targeting miR-3613-5p remains to be fully explored. Future studies should aim to validate these findings in larger human cohorts and investigate combination therapies targeting miR-3613-5p to enhance treatment efficacy and prevent the progression of CAG to gastric cancer. Such approaches could pave the way for novel clinical interventions and preventive strategies for gastric cancer.

## Conclusion

**FIGURE 7:**
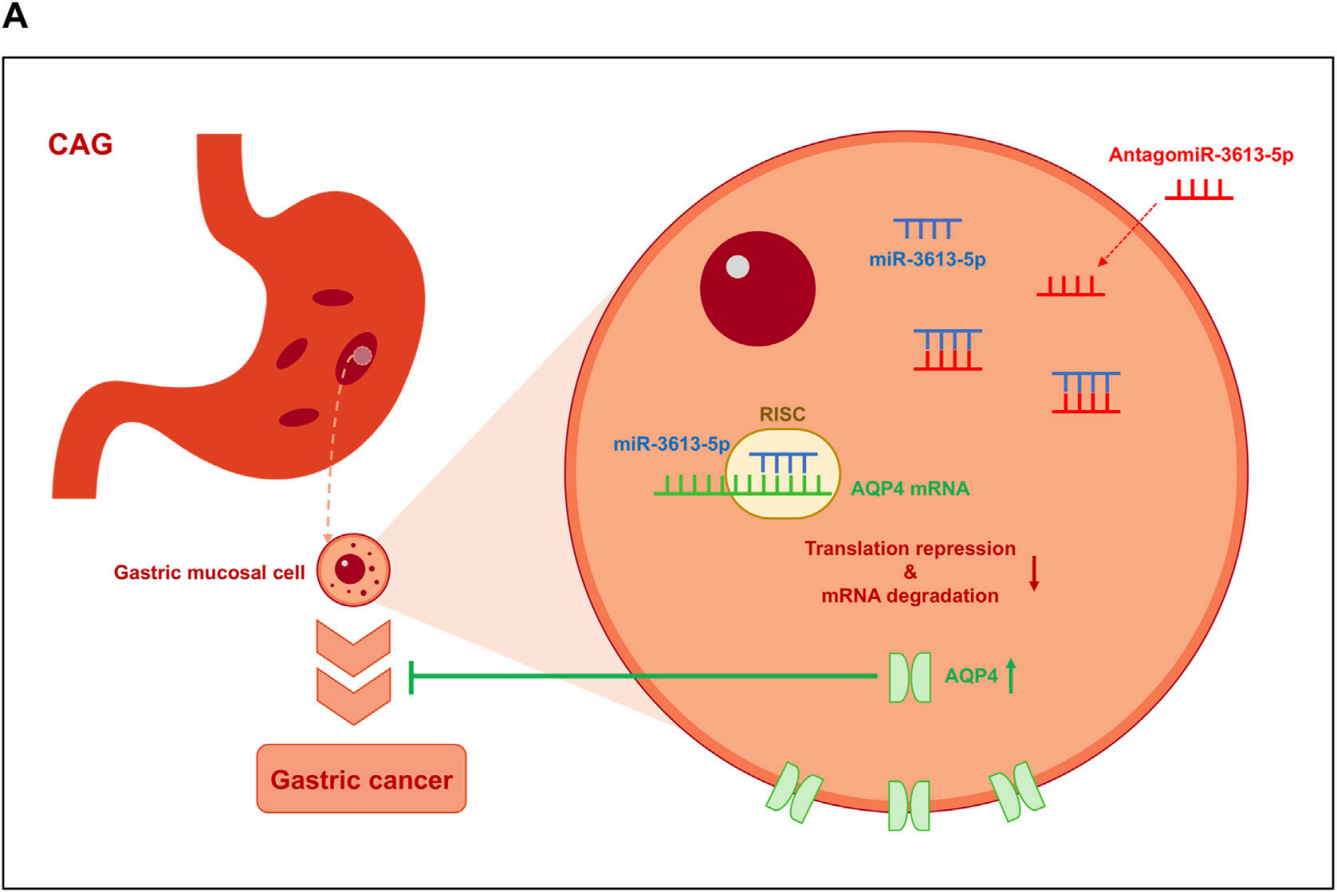
Model on how miR-3613-5p promotes the progression from CAG to gastric cancer. **(A)** miR-3613-5p inhibits the expression of the AQP4 gene by binding to its 3′ UTR, thereby promoting the progression from CAG to gastric cancer.

miR-3613-5p is significantly overexpressed in gastric mucosal tissue samples from CAG patients and in tumor samples from gastric cancer patients. High expression of miR-3613-5p was also observed in human gastric cancer cell lines and in the gastric mucosal of CAG mice. Overexpression of miR-3613-5p in gastric cancer cell lines promotes the proliferation and migration of gastric cancer cells, while silencing miR-3613-5p in CAG mice alleviates symptoms such as atrophy, hyperplasia, and inflammatory accumulation in the gastric mucosa. miR-3613-5p inhibits the expression of the AQP4 gene by binding to its 3′UTR, thereby promoting the progression from CAG to gastric cancer (Figure 7A).

## Data Availability

Experimental raw data are available at Figshare (DOI: 10.6084/m9.figshare.28632146). Bioinformatics analyses were performed using publicly available datasets from the GEO database. The processed data and scripts have been deposited in GitHub (https://github.com/YanXiu0105/AQP4-miR3613).

## Supplementary material

SUPPLEMENTARY FIGURE S1 Pearson correlation coefficient between AQP4 and miR-3613-5p. Data from The Cancer Genome Atlas (TCGA) database (https://www.cancer.gov/ccg/access-data).
