# Supplementary figures and images for: MiR-3613-5p targets AQP4 to promote the progression of chronic atrophic gastritis to gastric cancer
Source: Front Pharmacol. 2025 Apr 4;16:1523689. doi: 10.3389/fphar.2025.1523689 (PMC12006049; doi:10.3389/fphar.2025.1523689)

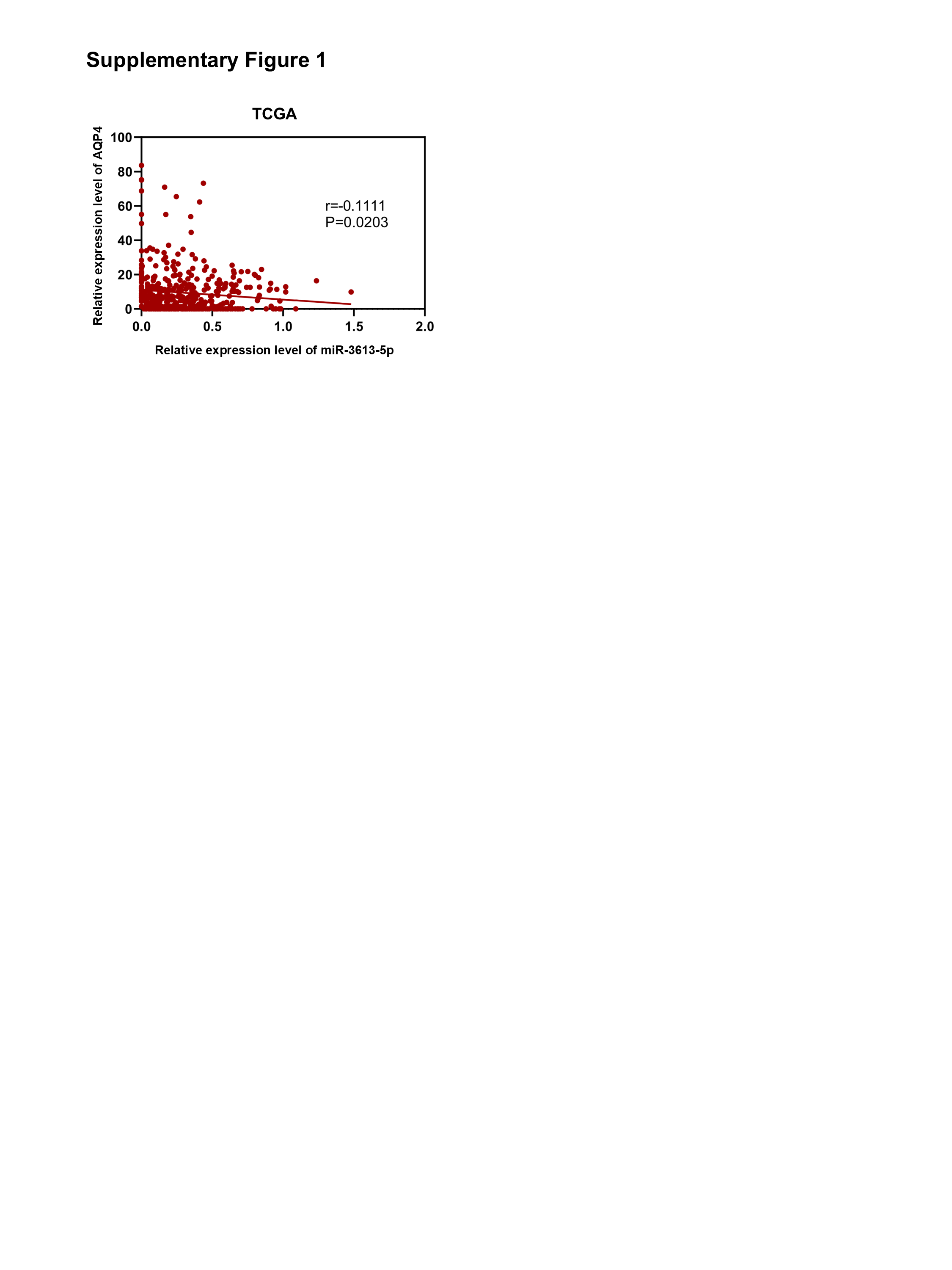

Supplement: Supplementary file 1 [file Image1.tif]
